# In-Depth Characterisation of Retinal Pigment Epithelium (RPE) Cells Derived from Human Induced Pluripotent Stem Cells (hiPSC)

**DOI:** 10.1007/s12017-014-8308-8

**Published:** 2014-05-07

**Authors:** Caroline Brandl, Stephanie J. Zimmermann, Vladimir M. Milenkovic, Sibylle M. G. Rosendahl, Felix Grassmann, Andrea Milenkovic, Ute Hehr, Marianne Federlin, Christian H. Wetzel, Horst Helbig, Bernhard H. F. Weber

**Affiliations:** 1 Institute of Human Genetics, University of Regensburg, Franz-Josef-Strauss-Allee 11, 93053 Regensburg, Germany; 2 Department of Ophthalmology, University Hospital Regensburg, Franz-Josef-Strauss-Allee 11, 93042 Regensburg, Germany; 3 Department of Psychiatry and Psychotherapy, Molecular Neuroscience, University of Regensburg, Franz-Josef-Strauss-Allee 11, 93053 Regensburg, Germany; 4 Department of Conservative Dentistry and Periodontology, University Hospital Regensburg, Franz-Josef-Strauss-Allee 11, 93042 Regensburg, Germany

**Keywords:** Induced pluripotent stem cell, Retinal pigment epithelium, Dermal fibroblast, Bestrophin-1, BEST1

## Abstract

Induced pluripotent stem cell (iPSC)-derived retinal pigment epithelium (RPE) has widely been appreciated as a promising tool to model human ocular disease emanating from primary RPE pathology. Here, we describe the successful reprogramming of adult human dermal fibroblasts to iPSCs and their differentiation to pure expandable RPE cells with structural and functional features characteristic for native RPE. Fibroblast cultures were established from skin biopsy material and subsequently reprogrammed following polycistronic lentiviral transduction with OCT4, SOX2, KLF4 and L-Myc. Fibroblast-derived iPSCs showed typical morphology, chromosomal integrity and a distinctive stem cell marker profile. Subsequent differentiation resulted in expandable pigmented hexagonal RPE cells. The cells revealed stable RNA expression of mature RPE markers RPE65, RLBP and BEST1. Immunolabelling verified localisation of BEST1 at the basolateral plasma membrane, and scanning electron microscopy showed typical microvilli at the apical side of iPSC-derived RPE cells. Transepithelial resistance was maintained at high levels during cell culture indicating functional formation of tight junctions. Secretion capacity was demonstrated for VEGF-A. Feeding of porcine photoreceptor outer segments revealed the proper ability of these cells for phagocytosis. IPSC-derived RPE cells largely maintained these properties after cryopreservation. Together, our study underlines that adult dermal fibroblasts can serve as a valuable resource for iPSC-derived RPE with characteristics highly reminiscent of true RPE cells. This will allow its broad application to establish cellular models for RPE-related human diseases.

**Electronic supplementary material:**

The online version of this article (doi:10.1007/s12017-014-8308-8) contains supplementary material, which is available to authorized users.

## Introduction

Induced pluripotent stem cells (iPSCs) have initially been established from mouse and subsequently from human cells (Takahashi and Yamanaka 2006; Takahashi et al. 2007), the latter demonstrating the successful reprogramming of human somatic cells into pluripotent cells resembling human embryonic stem cells (hESCs). Since then, human iPSCs (hiPSCs) have been appreciated as a promising source for disease modelling, drug screening and cell-based transplantation therapy in selected human degenerative diseases (Yamanaka 2009; Egashira et al. 2013; Okano et al. 2013).

Degenerative disorders involving the retinal pigment epithelium (RPE) are common in ocular diseases highlighting the crucial role of this post-mitotic cell layer in retinal homoeostasis (Strauss 2005; Singh et al. 2013a). Monogenic as well as complex RPE-related diseases, such as age-related macular degeneration, are common causes of blindness while therapeutic options are limited (Lim et al. 2012).

In vitro modelling of hiPSC-derived RPE cells has been a focus of research in ophthalmic research. Access to renewable sources of highly functional and expandable human RPE is ideal for studying these cells and the diseases affecting them (Du et al. 2011; Singh et al. 2013b). Different protocols for the differentiation of human pluripotent stem cells (hPSCs) into RPE have been described. Acquired cells were reported to display characteristic phenotypes as well as gene expression and exhibit key physiological functions (Ukrohne et al. 2012; Buchholz et al. 2013; Zhu et al. 2013; Rowland et al. 2013; Mekala et al. 2013; Maruotti et al. 2013; Singh et al. 2013a). RPE derived from hESCs has even reached the necessary standards for use in human clinical trials of cell-based transplantation therapy (Schwartz et al. 2012). Key parameters evaluated in many hPSC-RPE studies thus far include cell morphology, gene and protein expression, transepithelial resistance (TER) and photoreceptor outer segment (POS) phagocytosis (Buchholz et al. 2009; Ukrohne et al. 2012; Zhu et al. 2013; Singh et al. 2013a, b). Secretion capacities were investigated into fewer studies, describing polarised secretion of known biological factors like VEGF-A by hiPSC-RPE cells (Singh et al. 2013a). It has recently been shown that highly differentiated, pigmented hiPSC-RPE monolayers can undergo only limited serial expansions before losing key cytological and functional attributes due to replicative senescence (Singh et al. 2013a). Importantly, studies investigating cryostorability of hiPSC-RPE cells are scarce up to this point. Moreover, many protocols for RPE cell differentiation, if not the majority, use, e.g. neonatal foreskin fibroblasts, neonatal keratinocytes or even foetal lung fibroblast and foetal neural stem cells as a source, as it seems more feasible to reprogram and differentiate younger cells, due to their putatively higher plasticity (Ukrohne et al. 2012; Buchholz et al. 2013; Zhu et al. 2013; Maruotti et al. 2013).

In the present study, we report the successful reprogramming of human adult skin biopsy-derived fibroblasts to iPSCs and their differentiation to expandable and cryostorable hiPSC-RPE cells. Establishing and optimising such protocols will be particularly important for generating specific RPE cell models from patients with various diseases affecting the RPE. Our approach represents a further step towards establishing a hiPSC-RPE cell repository which yields pure populations of functional cells that display main features of native RPE. Characterisation of hiPSC-RPE cells during subcultivation has addressed structural and functional key aspects, the occurrence of replicative senescence and their cryostorability. We deepened morphological examinations by scanning electron microscopy. Furthermore, we emphasised protein expression and correct localisation of the RPE-specific marker BEST1 in hiPSC-RPE cells as a high-quality measure of proper differentiation.

## Materials and Methods

### Adult Human Dermal Fibroblasts

Biopsy material of healthy skin (4 mm) was obtained according to standard protocols from the Department of Dermatology, University Hospital Regensburg, Germany. Dermal tissue was cut into 0.5-mm pieces and subsequently seeded in 100-mm cell culture dishes in standard fibroblast growth medium (Dulbecco’s modified Eagle’s medium [DMEM] high-glucose with 4.5 g/l l-glutamine, supplemented with 10 % foetal bovine serum, 100 U/ml penicillin and 0.1 mg/ml streptomycin (all Gibco by Life Technologies, Darmstadt, Germany). After 10–15 days, an outgrowth of single fibroblastoid-shaped cells was observed. Cultures were maintained in humidified air (5 % CO_2_) at 37 °C, and medium was changed twice a week. After reaching confluency, cells were detached using trypsin–EDTA (Gibco by Life Technologies, Darmstadt, Germany) and subcultured in 75-cm^2^ cell culture flasks with fibroblast growth medium.

### Reprogramming and Expansion of hiPSC

Human iPSCs were obtained via polycistronic lentiviral transduction (Human STEMCCA Cre-Excisable Constitutive Polycistronic [OKS/L-Myc] Lentivirus Reprogramming Kit, Merck Millipore, Darmstadt, Germany) following the manufacturer’s recommendations. Briefly, human dermal fibroblasts (passage 4 or 5) were seeded in 6-well dishes at a density of 15,000 cells/well in fibroblast growth medium 1 day prior to infection. The following day, medium was changed and supplemented with polybrene (6 μg/ml, Merck Millipore, Darmstadt, Germany). Lentivirus was added with a multiplicity of infection (MOI) of 50, and spinfection was conducted at 800 g for 1 h. Medium was then changed, and fresh polybrene and lentivirus were added over night. After 5 days, infected fibroblasts were detached with trypsin–EDTA and replated on irradiated mouse embryonic fibroblasts (iMEFS, Merck Millipore, Darmstadt, Germany). Medium was switched to human embryonic stem cell (hESC) medium, containing DMEM/Ham’s F12, 2 mM l-glutamine, 20 % knockout serum replacement, 0.1 mM non-essential amino acids (all Gibco by Life Technologies, Darmstadt, Germany), 0.1 mM 2-mercaptoethanol (Merck Millipore, Darmstadt, Germany), 20 ng/ml bFGF (Pepro Tech, Hamburg, Germany), 50 mg/ml gentamicin (Sigma-Aldrich, Munich, Germany) and 0.1 % Human iPS Reprogramming Boost Supplement II (Merck Millipore, Darmstadt, Germany). Medium was changed every other day. Human iPSC colonies appeared between day 15 and 20 and were picked mechanically for monoclonal expansion under serum- and feeder-free conditions on hESC-qualified Matrigel (BD Biosciences, Heidelberg, Germany) in mTeSR™1 medium (Stemcell Technologies, Köln, Germany). Medium was changed daily. For propagation of hiPSCs, enzymatic passaging with dispase treatment (1 mg/ml, Stemcell Technologies, Köln, Germany) was applied every 4–5 days at splitting ratios of 1:3. For cryostorage at −190 °C, cells were kept in 90 % knockout serum replacement and 10 % DMSO (AppliChem, Darmstadt, Germany).

### RPE Differentiation and Cultivation

The protocol for RPE differentiation and cultivation was based on Ukrohne et al. (2012) with minor modifications. Human iPSCs were expanded to confluence in mTeSR™1 maintenance medium for 12–14 days. Differentiation was induced by switching hiPSC maintenance medium to RPE medium containing knockout DMEM, 2 mM l-glutamine, 20 % knockout serum replacement, 0.1 mM non-essential amino acids (all Gibco by Life Technologies, Darmstadt, Germany), 0.1 mM β-mercaptoethanol, 100 U/ml penicillin, 0.1 mg/ml streptomycin and 10 mM nicotinamide (Sigma-Aldrich, Munich, Germany). Medium was changed daily. Starting at week 4 of differentiation, 62 ng/ml activin A (Pepro Tech, Hamburg, Germany) was added to the medium until pigmented cell clusters appeared within the culture (within about 2 weeks). Consequently, clusters enlarged progressively during the following 2–3 weeks and were manually excised with a scalpel to enrich for iPSC-RPE cells. Pigmented patches were enzymatically dissected into single cells by Tryp-LE select (Gibco by Life Technologies, Darmstadt, Germany) and replated on growth factor-reduced (gfr-) Matrigel (dilution 1:30, BD Biosciences, Heidelberg, Germany)-coated cell culture plates in RPE medium without activin A. Within approximately 4 weeks, cells had reached confluence and had regained their characteristic morphology. They were then detached with 1 mM EDTA in PBS (phosphate-buffered saline, Gibco by Life Technologies, Darmstadt, Germany) for 10 min followed by Tryp-LE select for 10 min and split at ratios from 1:6 to 1:10 for subsequent expansion on gfr-Matrigel-coated plates in RPE medium without activin A. Medium was changed every other day, and passaging was performed every 4–5 weeks.

For further analysis, hiPSC-RPE cells were cultivated on 12-well or 6-well transwell filter inserts (0.4-µm pore size, Corning Costar by Sigma-Aldrich, Munich, Germany) coated with gfr-Matrigel at a dilution of 1:30. For cryostorage at −190 °C, cells were kept in CryoStor™CS10 medium (Stemcell Technologies, Köln, Germany).

### Reverse Transcription Polymerase Chain Reaction (RT-PCR) and Quantitative (q)RT-PCR

Total RNA was isolated from cultured cells using the RNeasy Mini Kit (Qiagen, Hilden, Germany) according to the manufacturer’s recommendations. On-column DNase digestion reliably removes genomic DNA contamination (Roche, Mannheim, Germany). First-strand cDNAs from 1 µg of total RNA were synthesised using the RevertAid H Minus First-Strand cDNA Synthesis Kit (Fermentas by Thermo Fisher Scientific, Schwerte, Germany).

For RT-PCR, 50 ng of cDNA was used as templates for PCR with Go Taq Polymerase (Promega, Mannheim, Germany) at a final volume of 25 μl. Gene-specific primers were designed online with the Primer3 programme (http://simgene.com/Primer3) and are given in Supplemental Table S1. PCR products were electrophoretically separated in a 2 % agarose gel. All genes were amplified for a total of 35 cycles, and the housekeeping gene glyceraldehyde-3-phosphate dehydrogenase (GAPDH) served as control for RNA integrity. Human iPSCs were further characterised with the hES/iPS Cell Pluripotency RT-PCR Kit (Applied Stem Cell, Menlo Park, USA).

For qRT-PCR analysis, first-strand cDNA was obtained from 2 µg total RNA using the high-capacity cDNA RT Kit with RNase Inhibitor (Life Technologies, Darmstadt, Germany). Expression of pluripotency markers and determination of trilineage differentiation potential were evaluated for hiPSCs with the TaqMan hPSC Scorecard™ Panel (384 well, ViiA7, Life Technologies, Darmstadt, Germany) following the company’s guidelines and using its cloud-based online analysis software.

### Immunofluorescence

For immunofluorescence analysis, hiPSCs were cultured on hESC-qualified Matrigel-coated glass chamber slides for 2–3 days in mTeSR™1 maintenance medium. Cells were washed twice with PBS, pH 7.4 and fixed with 4 % paraformaldehyde ([PFA] in PBS, Roth, Karlsruhe, Germany) for 10 min followed by three washing steps with PBS. To reduce unspecific background, cells were blocked for 30 min in PBS containing 0.3 % Triton X-100 (Sigma-Aldrich, Munich, Germany) and 10 % goat serum (Calbiochem, Darmstadt, Germany) at room temperature. Staining with primary antibody was performed in PBS supplemented with 0.1 % Triton X-100 and 2.5 % goat serum overnight at 4 °C. A list of primary antibodies and the respective dilutions are given in Supplemental Table S2. After three washing steps with PBS, secondary antibodies were added for 2 h at room temperature, together with a 1:1,000 dilution of 4′,6-diamidino-2-phenylindole (DAPI) dilactate (Sigma-Aldrich, Munich, Germany) for nuclei staining. As a negative control, each staining was carried out without the respective primary antibody (data not shown). In addition, to ensure specific of stem cell markers, human embryonic kidney 293 (HEK) cells were stained as negative controls (data not shown). After staining, cells were rinsed, mounted with DakoCytomation fluorescent mounting medium (DAKO, Hamburg, Germany) and viewed with a fluorescence microscope (Axioskop 2 mot plus, Zeiss, Göttingen, Germany).

Human iPSC-derived RPE cells cultured on either gfr-Matrigel-coated 12-well transwell filter inserts (0.4 µm pore) or gfr-Matrigel-coated glass cover slips were washed twice with PBS and fixed with 4 % PFA for 10 min from both sides of the transwell filter membrane. Reaction was stopped with 100 mM glycin (Merk, Darmstadt, Germany) in PBS. Cells were rinsed, and blocking solution (10 % normal goat serum, 0.2 % Triton X-100, 0.1 % purified BSA [Gibco by Life Technologies, Darmstadt, Germany], 0.05 % Tween 20 [AppliChem, Darmstadt, Germany], in PBS) was added for 1.5 h at room temperature. Primary antibodies and working dilutions are listed in Supplemental Table S2. After three washing steps in PBS buffer solution (containing 0.2 % Triton X-100, 0.1 % purified BSA, 0.05 % Tween 20), secondary antibodies (Supplemental Table S2) were incubated over night at 4 °C. Cells were rinsed with buffer solution for 20 min and with PBS only for 10 min at room temperature. Nuclei were stained with a 1:1,000 dilution of 4′,6-diamidino-2-phenylindole (DAPI) dilactate for 20 min. Cells were finally rinsed and mounted. Transwell filter membranes were excised with a scalpel and placed on object slides. For negative control, the procedure was carried out omitting the primary antibody (data not shown). Human iPSCs served as negative control and were stained according to this protocol to ensure RPE specificity of protein markers (data not shown). Cells were examined using confocal microscope LSM510 (Zeiss, Göttingen, Germany). Images were recorded at intensity levels below saturation, estimated by intensity analysis module.

### Surface Biotinylation, SDS Page and Western Blot Analysis

For biotinylation of plasma membrane proteins, a fresh solution of EZ-Link Sulfo-NHS-SS-Biotin (Thermo Fisher Scientific, Schwerte, Germany) was prepared at a concentration of 1 mg/ml in ice-cold PBS. Human iPS-derived RPE cells grown on 6-well transwell filters for 3 months were washed 3 times with ice-cold PBS. Biotin/PBS solution was added to the basal and the apical chamber followed by incubation for 30 min in the cold. Biotin reaction was stopped by adding 100 µl of stop solution (Life Technologies, Darmstadt, Germany) and extensive washing with ice-cold PBS.

Cells from a single 6-well transwell filter were collected in 1 ml lysis buffer (50 mM Tris, pH 7.5, 5 mM EDTA, 15 mM NaCl, 1 % Triton, 1 × Protease inhibitor cocktail (#04693116001, Roche, Mannheim, Germany) and homogenised by passing the lysate 8 times through a 25-gauge needle attached to a syringe. After incubation for 30 min at 4 °C, lysates were centrifuged for 15 min at 13,000 g. Streptavidin beads (100 µl, Life Technologies, Darmstadt, Germany) were added to the supernatant and incubated on a rotating wheel over night at 4 °C. Beads were washed 5 times with cold lysis buffer, and biotinylated proteins were eluted by boiling the sample for 5 min at 95 °C in SDS sample buffer containing 50 mM Tris-HCl pH 6.8, 2 % SDS, 10 % glycerol, 1 % β-mercaptoethanol, 12.5 mM EDTA and 0.02 % bromophenol blue. Proteins were separated by SDS-polyacrylamide gel electrophoresis on 10 % gels and subsequently transferred onto PVDF membranes (Merck Millipore, Darmstadt, Germany). After blocking in PBS (pH 7.4) containing 4 % non-fat dry milk, membranes were incubated with primary antibodies at 4 °C overnight (mouse anti-human bestrophin-1 monoclonal antibody [E6-6, ab2182, Abcam, Cambridge, UK], dilution 1:2,500, and mouse monoclonal antibody against β-actin [#5441, Sigma-Aldrich, Munich, Germany], dilution 1:10,000). Washing steps were performed with PBS containing 0.05 % Tween-20, followed by incubation with a 1:10,000 dilution of HRP-conjugated secondary antibody (Calbiochem/Merck, Darmstadt, Germany) for 2 h at room temperature. After a final washing with PBS containing 0.05 % Tween-20, protein labelling was visualised by enhanced chemiluminescence (ECL) using AGFA medical X-ray film (AGFA, Köln, Germany).

### Scanning Electron Microscopy (SEM)

For detailed visualisation, hiPSC-RPE cells were viewed both in high-vacuum (high-vac) and low-vacuum (low-vac) SEM mode. Human iPSC-RPE cells cultured on transwell filters for 3 months were rinsed once with PBS and fixed for 30 min at room temperature with 2.5 % glutaraldehyde (Serva, Heidelberg, Germany) in 0.1 M Soerensen buffer, pH 7.4 (Merck, Darmstadt, Germany) and aqua bidest. Then, cells were washed 3 times with 0.1 M Soerensen buffer, pH 7.4 for 5 min at room temperature.

For low-vac SEM, fixated cells were rinsed with aqua bidest for salt removal, brought on stubs via Leit-Tabs (Science Services, Munich, Germany) and analysed in moist condition with the FEI Quanta 400 FEG (FEI Deutschland GmbH, Kassel, Germany) in low-vac mode (LFD + EDX-PLA; 4.0 kV, Spot 4, WD ≈ 10 mm, 1.5 Torr, tilt 30°).

For high-vac SEM, fixated cells underwent serial dehydration via increasing ethanol concentrations (30–96 % EtOH). After treatment with the critical point dryer Balzers CPD 030 (BAL-TEC AG, Leica, Wetzlar, Germany), and positioning on stubs via Leit-Tabs (Science Services, Munich, Germany), cells were sputtered with platinum twice for 30 s at 30 mA in 50 mm distance (BAL-TEC SCD 005, BAL-TEC AG, Leica, Wetzlar, Germany). Cells were kept in the exsiccator and investigated in dry condition with the FEI Quanta 400 FEG in high-vac mode (4.0 kV, Spot 3, WD ≈ 6 mm, ≤10^−4^ Torr, tilt 30°).

### Chromosome Preparation and Karyotyping

For numerical and structural chromosome analyses of dermal fibroblasts and hiPSCs, high-resolution karyotyping was performed. Fibroblasts were grown in T25 culture flasks in fibroblast growth medium. Human iPSCs were cultivated in T25 flasks coated with hESC-qualified Matrigel in mTeSR™1 maintenance medium. Cells were synchronised using thymidine solution (Sigma-Aldrich, Munich, Germany) and subsequently treated with colcemid (Roche, Mannheim, Germany) for 10 min at 37 °C. After detachment with trypsin–EDTA, cells were centrifuged, and the cell pellet was re-suspended and maintained in hypotonic solution (75 mM KCl) for 12 min at 37 °C. Cells were then fixed in methanol and acetic acid. Metaphase spreads were prepared on cover slips, dried overnight and Giemsa stained (Sigma-Aldrich, Munich, Germany) after trypsin pre-treatment.

### Transepithelial Resistance (TER) Measurements

For functional assessment, TER of hiPSC-RPE monolayers cultured on gfr-Matrigel-coated transwell filters was measured using an epithelial voltohmmeter (EVOMX) following the manufacturer’s instructions (World Precision Instruments, Berlin, Germany). Briefly, electrodes were sterilized with 70 % ethanol, rinsed in 150 mM NaCl solution and placed in the transwell filter with the longer voltage electrode positioned in the lower chamber touching the bottom of the dish while the shorter current electrode was placed in the upper chamber. Net TER was calculated from TER recordings by subtracting background measurements obtained from gfr-Matrigel-coated transwell filters without cells. TER measurements were obtained by multiplying net TER values with the surface area of the transwell filters and reported as Ω*cm^2^.

### VEGF Secretion

To determine secretion properties, hiPSC-RPE monolayers were grown for 2 months on gfr-Matrigel-coated 12-well transwell filters. Apical and basal VEGF-A levels were measured using the commercially available Human VEGF Quantikine ELISA Kit (#DVE00, R&D Systems, Wiesbaden-Nordenstadt, Germany) in accordance with the manufacturer’s instructions. In brief, 24-h-old extracellular RPE medium was taken from the apical and basal chamber. Microplate reading was performed with the FLUOstar OPTIMA (BMG Labtech, Ortenberg, Germany). Net VEGF-A was calculated by subtracting unused medium (negative control).

### Photoreceptor Outer Segment (POS) Phagocytosis

Human iPSC-RPE cells were fed porcine POS which were obtained from porcine retinae after homogenisation with 20 % sucrose (Merck, Darmstadt, Germany) and 1 x Protease inhibitor cocktail. After centrifugation for 5 min at 500 g in 4 °C, the supernatant was again homogenised with 20 % sucrose and protease inhibitor and consequently separated by density gradient centrifugation (100,000 g, 120 min, 4 °C, without brake). The fraction containing POS was transferred into 11-ml tubes (BD Biosciences, Heidelberg, Germany), rinsed with 0.02 M Tris buffer and centrifuged at 10,000 g for 10 min without brake. The pellet was re-suspended in cryosolution containing 10 mM phosphate buffer, 0.1 mM NaCl and 2.5 % sucrose. POS were counted using the Neubauer counting chamber, aliquoted and stored at −80 °C.

Prior to feeding, POS were labelled with Oregon green 488 (OG488, Molecular probes by Life Technologies, Darmstadt, Germany). POS were thawed, pelleted (10,000 g, 3 min, 4 °C) and re-suspended in 100 µl 0.1 M bicarbonate buffer, pH 8.3. After adding 10 µl OG488 in 1:100 diluted DMSO, POS were vortexed and incubated on a shaker for 90 min in the dark at 4 °C. After two washing steps with PBS, the cells were pelleted at 10,000 g and blocked with serum-containing medium on a shaker for 20 min in the dark at 4 °C. Then, POS were centrifuged (10,000 g, 3 min, 4 °C), and the labelled POS were re-suspended in fresh Opti-MEM (Gibco by Life Technologies, Darmstadt, Germany).

To investigate phagocytotic uptake and degradation in a timeline experiment, hiPSC-RPE cells cultured on transwell filters for 2 months were incubated with labelled POS in RPE medium, applying a concentration of 20 POS per cell for 90 min, 6 and 24 h. Cryostored hiPSC-RPE cells cultured on transwell filters for 2 months were incubated with 20 POS per cell for 24 h. Feeding experiments were analysed by confocal microscopy. Briefly, cells were thoroughly washed with PBS to remove non-incorporated POS and immunostained with rabbit anti-human ZO-1 polyclonal antibody (#617300, Zymed Laboratories by Life Technologies, Darmstadt, Germany). Cells were subsequently mounted and examined using confocal microscope LSM510. Images were recorded at intensity levels below saturation, estimated by intensity analysis module. Confocal images were quantitatively analysed using an ImageJ software package (version 1.47v) following procedures described by Abramoff et al. (2004). For analysis of POS phagocytosis, confocal image files (z-stacks) taken with the green channel were loaded into ImageJ and submitted to Z-Project Sum Slices command. Next, threshold levels were determined by analysing the histogram of the entire image. An equal threshold level was used for all follow-up images. The total number of POS was counted using Analyze Particles command.

### Statistics

Data throughout the manuscript are expressed as mean ± standard deviation (SD). If compared using the unpaired Student’s *t* test, significance was reported for *p* values ≤0.05.

## Results

### Human iPSCs Derived from Adult Human Dermal Fibroblasts Reveal Chromosomal Integrity and Distinctive Stem Cell Properties

**Fig. 1:**
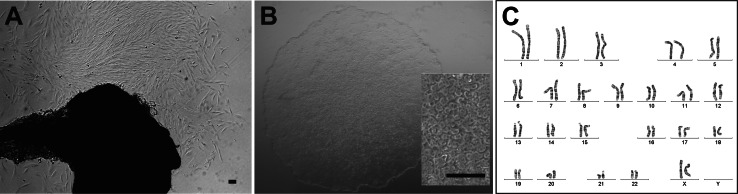
Morphology and chromosomal integrity of adult human dermal fibroblast-derived hiPSCs. **a** Outgrowth of human dermal fibroblasts from skin biopsy tissue obtained from a healthy 26-year-old female donor (“WT1”). **b** Fibroblast-derived hiPSC_WT1c1 at passage 9 show characteristic hESC-like morphology with round, sharp-edged colonies and tightly packed cells. Enlarged section: hiPSCs reveal prominent nucleoli with a high ratio of nucleus to cytoplasm volume. **c** Karyotype of hiPSC_WT1c1 at passage 9 shows a normal karyogram (46, XX). There are no structural or numerical aberrations detectable. *Scale bars* 100 µm

Skin biopsies from a total of five unrelated probands were taken in the course of this study. Here, we present an in-depth characterisation of a cell line derived from a 26-year-old healthy female donor (“WT1”). After 15 days in culture, dermal fibroblasts sprouted from the skin biopsy and were subcultured (Fig. 1a). At passage 5, reprogramming experiments were initiated with polycistronic lentiviral transduction. A total of five individual clones (named hiPSC_WT1c1 to c5) were subcultured in serum-free and feeder-free conditions for at least 35 passages. The hiPSCs showed typical hESC-like morphology (Fig. 1b), and there were no signs of increased differentiation or slower growth in higher passages. Karyotyping demonstrated normal karyotype for both fibroblast (passage 6, data not shown) and the hiPSC lines at passage 9 (Fig. 1c). At passage 21, hiPSCs revealed a mosaic with 47,XXX in one clone and a mosaic with trisomy 8 in a second clone (data not shown). Therefore, subsequent differentiation of hiPSCs was initiated before passage 10 to ensure chromosomal integrity.

**Fig. 2:**
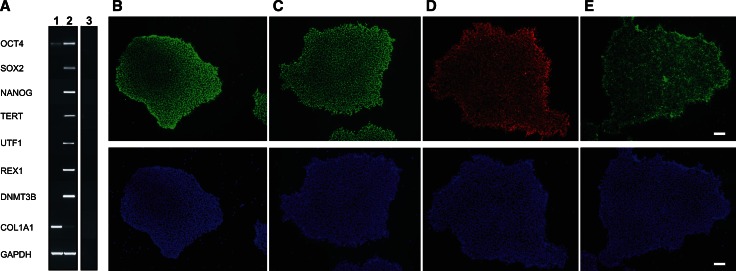
RNA and protein expression profiling of adult human dermal fibroblast-derived hiPSCs. **a** RT-PCR analyses expression of several typical stem cell markers (OCT4, SOX2, NANOG, TERT, UTF1, REX1, DNMT3B) and the fibroblast marker COL1A1 in total RNA derived from adult human dermal fibroblasts (*lane 1*) and hiPSC cell line hiPSC_WT1c1 (*lane 2*). RT-PCR was performed with gene-specific primers as given in Supplemental Table S1 and electrophoretically separated in a 2 % agarose gel. The housekeeping gene GAPDH was used to control for RNA integrity. Total RNA without reverse transcriptase served as negative control (*lane 3*). **b**–**e** For immunofluorescence analysis of stem cell marker expression, hiPSC_WT1c1 at passage 9 were stained against NANOG (*green*) (**b**), OCT4 (*green*) (**c**), SSEA3 (*red*) (**d**), TRA-1-60 (*green*) (**e**). Nuclei were stained with DAPI (*blue*, below). *Scale bars* 100 µm

RT-PCR and qRT-PCR experiments with hiPSC RNA showed an expression profile characteristic for stem cell markers (Fig. 2a, Supplemental Figure S1). For RT-PCR, hiPSCs was compared to its originating dermal fibroblast cell line (Fig. 2a). The iPSCs were positive for endogenous POU class 5 homeobox 1 (*OCT4*), SRY [sex determining region Y]-box 2 (*SOX2*), Nanog homeobox (*NANOG*), telomerase reverse transcriptase (*TERT*), undifferentiated embryonic cell transcription factor 1 (*UTF1*), zinc finger protein (*REX1*) and DNA (cytosine-5-)-methyltransferase 3 beta (*DNMT3B*). In contrast, fibroblast RNA was negative for the stem cell markers tested except for OCT4 which was weakly expressed. Collagen type I, alpha 1 (*COL1A1*) was strongly expressed in fibroblasts and showed a minor expression in hiPSCs while housekeeping gene glyceraldehyde-3-phosphate dehydrogenase (*GAPDH*) was strongly expressed in both cell lines.

The TaqMan hPSC Scorecard Panel evaluates pluripotency and detects germ layer bias by providing a pre-manufactured qRT-PCR assay and special cloud-based data analysis software. Analysing hiPSC RNA, pluripotency marker expression was comparable to the reference standard given in the analysis software (Supplemental Figure S1). Regarding germ layer markers, a comparison with standard hiPSC lines revealed downregulated expression for endoderm markers (1.5-fold) and highly significant for ectoderm markers (2.69-fold). Mesoderm markers were slightly downregulated but not significantly altered to standard (0.36-fold) (Supplemental Figure S1).

Immunofluorescence labelling of hiPSC colonies revealed expression of the four key pluripotency markers including *OCT4*, *NANOG*, *SSEA3* and *TRA*-*1*-*60* (Fig. 2b–e). Nuclei were positively stained with DAPI (blue). In contrast, HEK 293 cells serving as negative control showed no expression of stem cell markers (data not shown).

### RPE Differentiation into Pure and Expandable hiPSC-RPE Cells

**Fig. 3:**
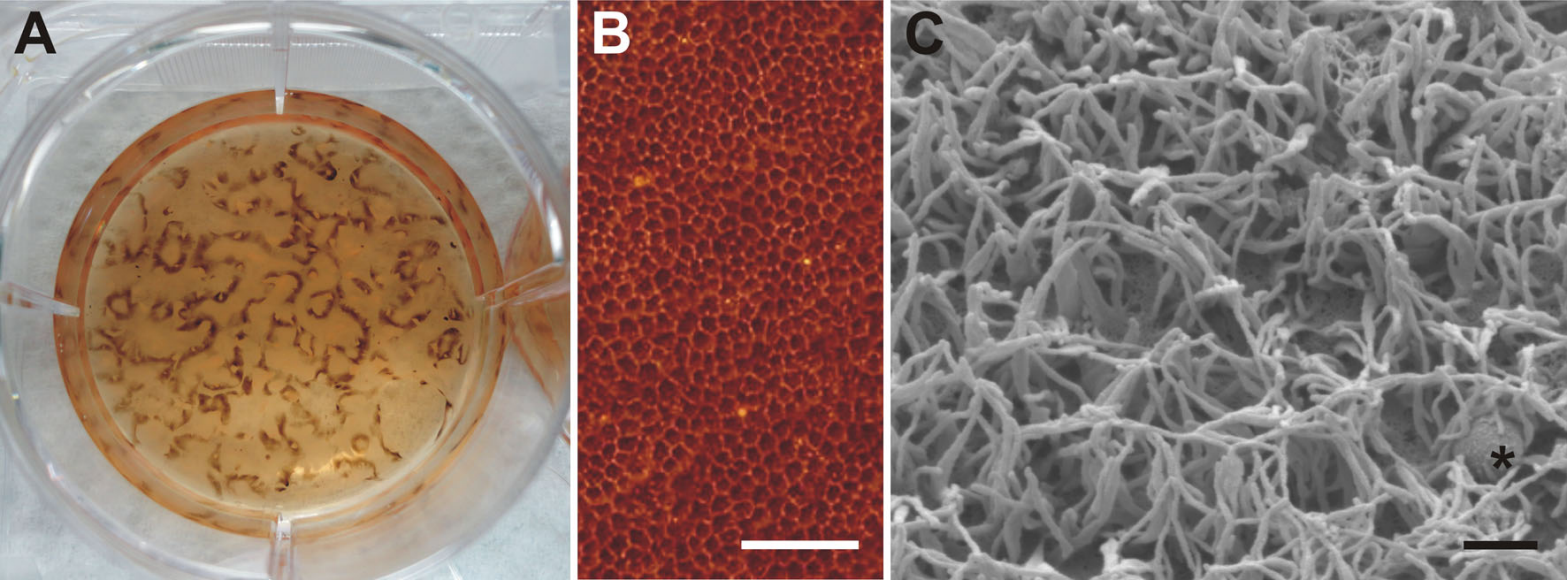
Morphology of hiPSC-RPE cells. **a** In cell line, hiPSC-RPE_WT1c1 pigmented cell clusters appear within 8 weeks after induction of RPE differentiation in hESC-qualified Matrigel-coated 6-well culture plates. **b** The pigmented cells were investigated via inverted light microscope and show a typical hexagonal morphology. *Scale bar* 100 µm. **c** Scanning electron microscopy (SEM) in high-vac mode reveals characteristic, apical microvilli. Note the vesicle-like structure (*asterisk*). *Scale bar* 1 µm

About 8 weeks after induction of RPE cell differentiation, pigmented clusters of hexagonal cells were visible (Fig. 3a, b). Human iPSC-RPE cells were subcultured both on gfr-Matrigel-coated cell culture plates and gfr-Matrigel-coated transwell filters. After 6 weeks on culture plates, conditions for hiPSC-RPE cells seemed less favourable when compared to transwell filters where cells could be grown for 6 months without passaging (data not shown). The iPSC-RPE lost pigmentation after initial passaging, which usually returned during the following 4–6 weeks. In two of the five cell lines analysed pigmentation never returned.

### Human iPSC-RPE Cells Demonstrate High-Quality, High-Purity and Adequate RPE Marker Expression

To analyse hiPSC-RPE cell morphology, cell culture preparations were viewed both in high-vac and low-vac scanning electron microscopy mode. SEM of hiPSC-RPE grown on transwell filter revealed the typical hexagonal cell shape (moist condition, low vac, data not shown) and the presence of characteristic microvilli at the apical side of the cells (dry condition, high vac, Fig. 3c).

**Fig. 4:**
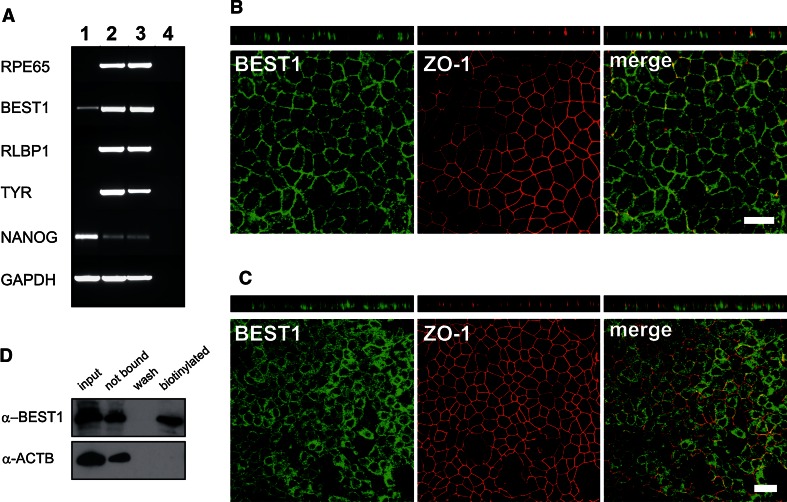
Expression profiling of hiPSC-RPE cells. **a** RNA expression profiling was done for mature RPE markers (*RPE65*, *BEST1*, *RLBP1*), the melanogenesis marker *TYR* and the stem cell marker *NANOG* in total RNA derived from hiPSCs (*lane 1*), hiPSC-RPE (*lane 2*) and native RPE (*lane 3*). RT-PCR was performed with gene-specific primers as given in Supplemental Table S1 and electrophoretically separated in a 2 % agarose gel. The housekeeping gene GAPDH was used to control for RNA integrity. Total RNA without reverse transcriptase served as negative control (*lane 4*). **b** Antibody labelling against RPE-specific marker BEST1 (*green*) and tight junction marker zonula occludens 1 (ZO-1, *red*). Endogenous BEST1 is targeted to the basolateral plasma membrane, whereas ZO-1 shows apical localisation. Shown are *x*–*y* and *x*–*z* projections of monolayers of polarised hiPSC-RPE_WT1c1, passage 4, grown on transwell filters for 2 months. *Scale bars* 20 µm. **c** Immunofluorescence stainings against BEST1 (*green*) and ZO-1 (*red*) in hiPSC-RPE cells cultured unpolarised on glass cover slides. Endogenous BEST1 protein localises less efficiently to the basolateral plasma membrane, whereas ZO-1 shows the typical apical localisation. Shown are *x*–*y* and *x*–*z* projections of monolayers of unpolarised hiPSC-RPE_WT1c1, passage 4, grown on glass cover slips for 2 months. *Scale bars* 20 µm. **d** Cell surface expression of BEST1 was shown by surface protein biotinylation, followed by precipitation with streptavidin. Immunoblot analysis with BEST1 monoclonal antibody demonstrates a substantial staining in the biotinylated cell membrane fraction (biotin). Co-staining with anti beta-actin (ACTB) served as negative control

An RNA expression profile for characteristic mature RPE markers was established by RT-PCR for hiPSC-RPE cells in comparison with its originating hiPSCs and native human RPE (Fig. 4a). Results showed strong expression of retinal pigment epithelium-specific protein 65 kDa (*RPE65*), bestrophin-1 (*BEST1*), retinaldehyde-binding protein 1 (*RLBP1*) and the melanogenesis marker tyrosinase (*TYR*) in hiPSC-RPE cells and native RPE, but no or only weak expression for *BEST1* in hiPSCs. Stem cell marker *NANOG* was weakly present in differentiated hiPSC-RPE and in native human RPE but showed a strong expression in hiPSCs.

Immunostaining demonstrated the presence of RPE-specific marker proteins BEST1 and tight junction marker zonula occludens 1 (ZO-1) (Fig. 4b, c). Whole 12-well filter inserts were stained and in total analysed under the microscope. Every single cell in the monolayer culture was positively stained for these 2 markers, demonstrating 100 % purity of the hiPSC-RPE cell culture with homogeneous staining patterns. When polarised on transwell filters for 2 months at passage 4, confocal microscopy verified localisation of BEST1 protein at the basolateral plasma membrane and ZO-1 at the apical side of hiPSC-RPE cells (Fig. 4b). When hiPSC-RPE cells were cultured unpolarised on glass cover slips for 2 months at passage 4, BEST1 protein was found mostly in the cytosol and less efficiently localised to the plasma membrane (Fig. 4c). Also, gaps in the monolayer due to cell loss (data not shown) were more often observed in unpolarised versus polarised hiPSC-RPE cells (Fig. 4b, c). As a negative control, hiPSC were also stained against BEST and ZO-1 protein and revealed no expression of the RPE markers (data not shown).

To determine whether BEST1 protein is truly present at the plasma membrane, surface protein biotinylation was performed to label plasma membrane proteins in hiPSC-RPE cells. Immunoblot analysis showed a substantial staining of BEST1 protein in the biotinylated fraction (Fig. 4d). An antibody against the intracellular protein beta-actin (ACTB) served as negative control and revealed no signal. Interestingly, hiPSC-RPE cells at passage 6 after 2 months of cultivation on transwell filters also revealed BEST1- and ZO-1-positive cells, but BEST1 was found diffusely in the cytosol and less efficiently localised to the plasma membrane (data not shown).

### Human iPSC-RPE Cells Demonstrate Key RPE Functions

**Fig. 5:**
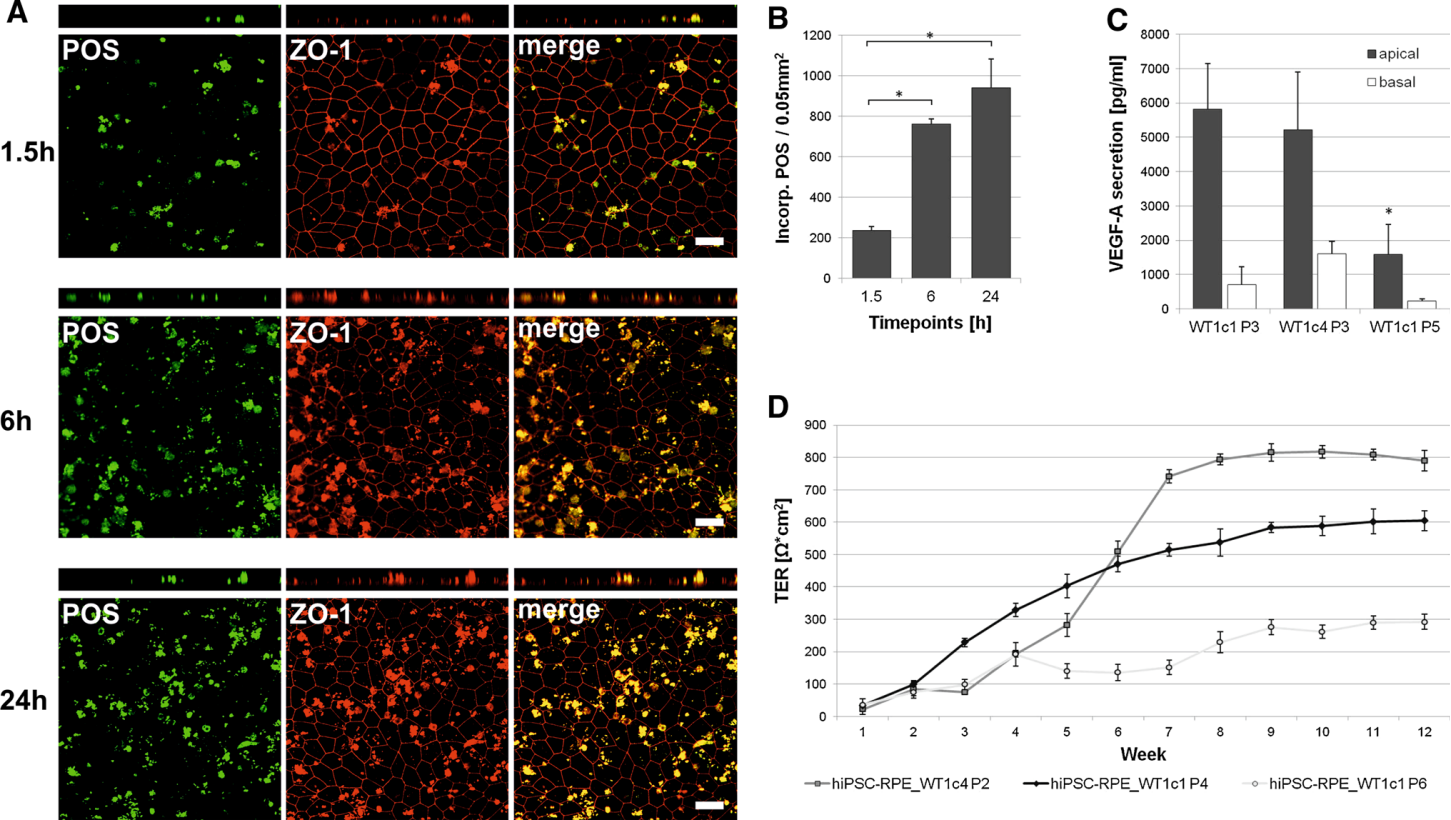
Functional characterisation of hiPSC-RPE cells. **a** Analysis of photoreceptor outer segment (POS) phagocytosis. Human iPSC-RPE cells were incubated with Oregon green-labelled POS for 1.5, 6 and 24 h (about 20 POS per cell). ZO-1 apical staining (*red*) shows relative localisation of the incorporated and partially digested POS. Shown are *x*–*y* and *x*–*z* projections of monolayers of hiPSC-RPE_WT1c1, passage 4, grown on transwell filters. *Scale bars* 20 µm. Due to known autofluorescence of POS, they are also visible in the red channel. **b** Quantitative analysis of POS phagocytosis. Incorporated POS were automatically counted in confocal images for the three time points. *Each bar* represents the average ± SD of six images, each covering an area of 0.05 mm^2^ (* = Student’s *t* test: *p* ≤ 0.05). **c** Measurement of apical and basal VEGF-A secretion in hiPSC-RPE cell lines at different passages. VEGF-A secretion is calculated as net VEGF-A levels in pg/ml. Each bar represents the average ± SD of at least three biological replicates (* = Student’s *t* test: *p* ≤ 0.05). **d** Measurement of transepithelial resistance (TER) in hiPSC-RPE cell lines tested at various passages. TER is calculated as net Ω*cm^2^. *Each data point* represents the average ± SD of at least three biological replicates

To address a fundamental aspect of RPE cell function, Oregon green-labelled POS were fed in a timeline experiment (1.5, 6, 24 h) at a concentration of 20 POS per cell to hiPSC-RPE cells cultured on transwell filters for 2 months. Phagocytotic uptake and degradation activity was monitored by confocal microscopy (Fig. 5a). In addition, incorporated POS were recorded automatically in the confocal images for quantitative follow-up (Fig. 5b). After 1.5 h, POS became incorporated by hiPSC-RPE cells (236 POS signals ±21.20 SD per 0.05 mm^2^) (Fig. 5a). Quantitative evaluation revealed a dramatic increase in phagocytotic activity from 1.5 to 6 h (762 POS signals ±26.51 SD per 0.05 mm^2^; *p* = 8.63E−07) which slowed at 24 h (941 POS signals ±143.07 SD per 0.05 mm^2^; *p* = 0.10, Fig. 5b). POS generally reveal a defined size (approx. 4–6 µm in diameter) and a smooth, roundish shape. At 24 h, more POS appeared partly digested and degraded when compared to the 1.5- and 6-h time points (Fig. 5a).

To elucidate whether hiPSC-RPE cells are capable of secretion of known biomolecules, levels of VEGF-A were measured in the apical and basal extracellular media of hiPSC-RPE cells grown on transwell filter inserts for 2 months. VEGF-A was predominantly secreted into the apical media compartment (hiPSC-RPE WT1c1 P3: 5,810 pg/ml ±1,339.78 SD) and showed a marked decline with increased passaging (hiPSC-RPE WT1c1 P5: 1,593 pg/ml ±873.69 SD; *p* = 0.0083) (Fig. 5c).

As a third aspect of RPE function, transepithelial resistance (TER) was determined in hiPSC-RPE cells cultured on transwell filters. Weekly measurements were taken over a 3-month period. Net TER demonstrated a significant increase during the first 6–8 weeks for hiPSC-RPE_WT1c4 at passage 2 (Fig. 5d). TER was maintained at high levels in this cell line for at least another 12 weeks. With increasing passages of hiPSC-RPE cells from passage 2 to passage 6, there was decline of TER (Fig. 5d). For all cell lines analysed, there was a significant loss of TER to below 300 Ω*cm^2^ after 24 weeks of cultivation (data not shown).

### Human iPSC-RPE Cells Largely Maintain Key Structural and Functional Aspects After Cryopreservation

For generation of cell repositories, cryostorage of hiPSC-RPE cells would be ideal. Therefore, key structural and functional aspects of cryopreserved hiPSC-RPE cells were analysed. After thawing, cells expanded and developed monolayers with characteristic hexagonal morphology (data not shown).

**Fig. 6:**
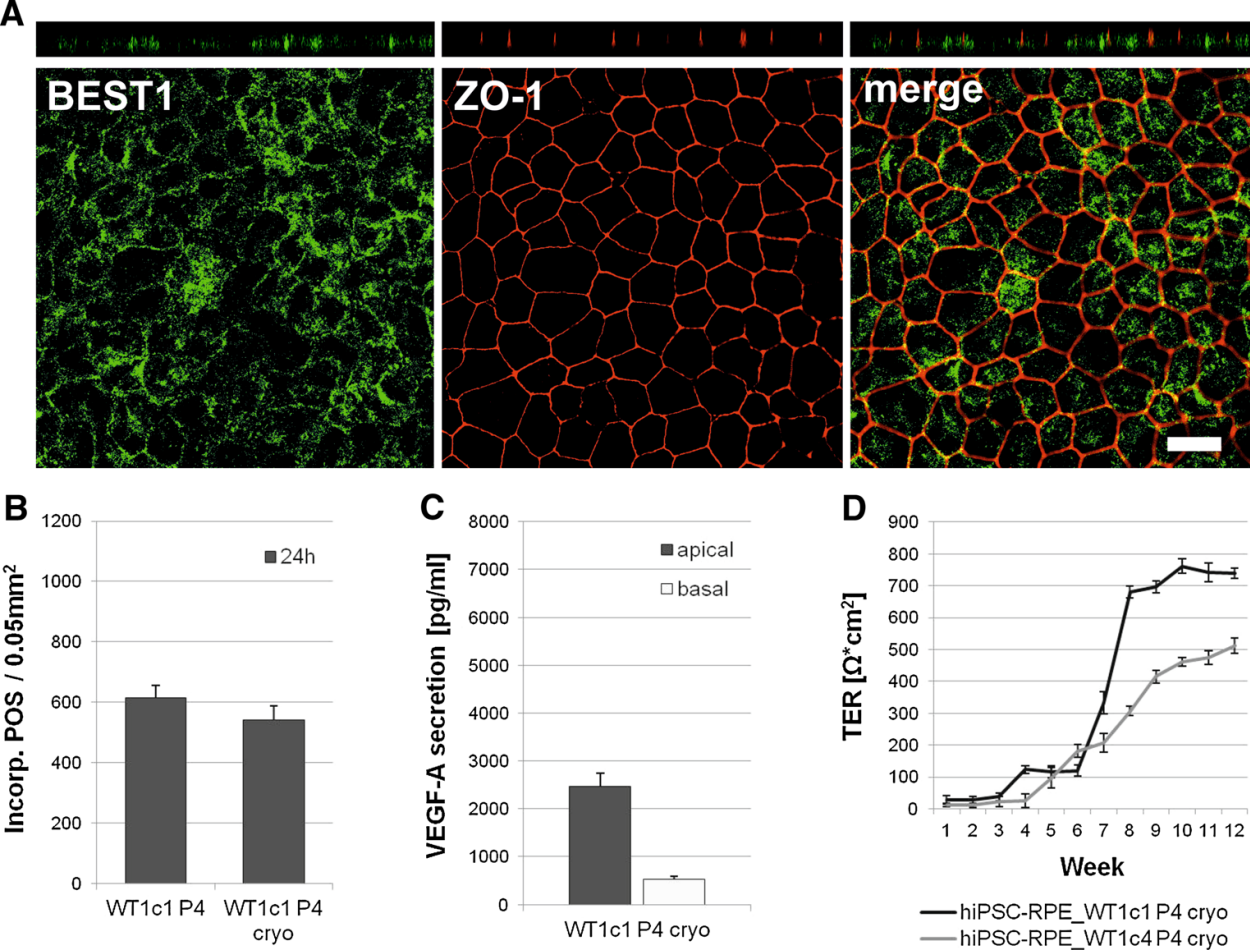
Structural and functional characterisation of hiPSC-RPE cells after cryopreservation. **a** Antibody labelling against RPE-specific marker BEST1 (*green*) and tight junction marker zonula occludens 1 (ZO-1, *red*). Endogenous BEST1 is targeted less efficiently to the basolateral plasma membrane, whereas ZO-1 shows apical localisation. Shown are *x*–*y* and *x*–*z* projections of monolayers of polarised hiPSC-RPE_WT1c1, passage 4, after cryostorage, grown on transwell filters for 10 weeks. *Scale bars* 20 µm. **b** Quantitative analysis of POS phagocytosis. Incorporated POS were automatically counted in confocal images from continuously cultured hiPSC-RPE WT1c1 passage 4 and cryostored hiPSC-RPE WT1c1 passage 4. *Each bar* represents the average ± SD of six images, each covering an area of 0.05 mm^2^ (* = Student’s *t* test: *p* ≤ 0.05). **c** Measurement of apical and basal VEGF-A secretion in hiPSC-RPE cell lines after cryostorage. VEGF-A secretion is calculated as net VEGF-A levels in pg/ml. *Each bar* represents the average ± SD of at least three biological replicates. **d** Measurement of transepithelial resistance (TER) in hiPSC-RPE cell lines tested after cryostorage. TER is calculated as net Ω*cm^2^. *Each data point* represents the average ± SD of at least three biological replicates

When cryostored hiPSC-RPE cells were grown polarised on transwell filters for at least 2 months, confocal microscopy verified BEST1 protein expression, but less efficiently localised to the basolateral plasma membrane (Fig. 6a). ZO-1 was detectable at the apical side of cryostored hiPSC-RPE cells (Fig. 6a).

Phagocytotic properties of cryostored hiPSC-RPE cells were directly compared to continuous hiPSC-RPE cells. Therefore, POS at a concentration of 20 POS per cell were fed to both continuous and cryostored hiPSC-RPE cells cultured on transwell filters for 10 weeks. Uptake and degradation activity was monitored after 24 h by confocal microscopy. Incorporated POS were recorded automatically in the confocal images for quantitative analysis (Fig. 6b). Quantitative evaluation of POS feeding revealed no statistically significant differences in phagocytotic properties after 24 h (hiPSC-RPE WT1c1 P4: 613.67 POS signals ±41.49 SD per 0.05 mm^2^; hiPSC-RPE WT1c1 P4 cryo: 542.34 POS signals ±46.92 SD per 0.05 mm^2^; *p* = 0.12) (Fig. 6b).

VEGF-A secretion was measured in the apical and basal extracellular media of cryostored hiPSC-RPE cells grown on transwell filter inserts for 10 weeks. VEGF-A was predominantly secreted into the apical media compartment (Fig. 6c).

TER measurements of cryopreserved hiPSC-RPE cell lines at passage 4 were performed weekly and observed a strong increase during weeks 6–9 (Fig. 6d). TER was maintained at high levels in this cell lines for another 8 weeks.

## Discussion

In the present study, we demonstrated the successful reprogramming of adult human dermal fibroblasts into iPSCs via polycistronic lentiviral overexpression of transcription factors OCT4, SOX2, KLF4 and L-Myc. The iPSCs developed characteristic hESC-like morphology including round, sharp-edged colonies with tightly packed cells, and prominent nucleoli with a high ratio of nucleus to cytoplasm volume (Takahashi et al. 2007). The iPSC morphology was used as a basic indicator to initiate reprogramming and subcultivation. Additional RNA and protein profiling revealed distinctive stem cell marker properties. This is generally an accepted approach to characterise the pluripotency of a stem cell line. The weak RNA expression of endogenous OCT4 frequently found in our dermal fibroblast cell cultures is unexplained and, so far, has not been reported in the literature. The faint expression of COL1A1 in the hiPSCs could be due to re-differentiation of a few cells and appears to be a regular observation (Takahashi and Yamanaka 2006; Takahashi et al. 2007).

For a quick and comprehensive analysis of pluripotency in the hiPSC lines, we used a commercial panel of specific, well-known markers relative to a reference standard (Bock et al. 2011). This includes genome-wide reference maps of DNA methylation and gene expression for 20 previously derived human ES cell lines and 12 human iPS cell lines. In addition, the *in vitro* differentiation potential of these cell lines was determined (Bock et al. 2011). Using this tool, we show pluripotency of our hiPSC lines while the germ layer markers indicate downregulation of endoderm and especially ectoderm markers. So far, we have not evaluated differentiation of our hiPSC lines into other germ layers than the RPE which arises ontogenetically from the neuroectoderm. The possibility that an altered germ layer profile as determined in our experiment could influence the effectiveness and quality of hiPSCs to differentiate in certain cell types is unclear at present and needs systematically to be explored.

Monitoring of genomic integrity of hiPSCs is crucial, particularly at early passages (Mayshar et al. 2010). It is not uncommon that hiPSCs derived from fibroblasts develop numerical or structural aberrations, limiting the utility of such genetically unstable cell lines (Mayshar et al. 2010). Chromosomal aberrations could affect the differentiation capacity of the cell line and are likely to influence the interpretation of functional studies with differentiated cells derived from genomically altered hiPSCs. Previous studies show that hiPSCs can acquire chromosomal abnormalities upon prolonged time in culture (Mayshar et al. 2010). We made similar observations and found chromosomal aberrations at passages higher than 20. However, studies also reported aberrations present in early passages with or without apparent somatic cell origin (Mayshar et al. 2010). Therefore, we used early passage hiPSCs for differentiation experiments, but not without testing their chromosomal stability and the integrity of their fibroblast origin.

Several protocols for RPE cell differentiation have been described (Ukrohne et al. 2012; Buchholz et al. 2013; Zhu et al. 2013; Rowland et al. 2013; Maruotti et al. 2013; Singh et al. 2013b). We have slightly modified an earlier protocol by Ukrohne et al. (2012) which proofed highly efficient. Within 8 weeks, 25–40 differently sized pigmented cell clusters appeared within one 6-well of confluent hiPSC colonies. By mechanically collecting these pigmented clusters, we generally obtained homogenous monolayer of pure RPE cells already after a single passage. Only rarely did we find non-RPE, more fibroblast-shaped cells after initial passaging. Interestingly, these cells were no longer detectable after subsequent passages. Contamination from undifferentiated hiPSCs is of particular concern, since these cells can, by definition, proliferate and form teratomas in host tissue (Schwartz et al. 2012). In our hands, coating cell culture surfaces with growth factor-reduced Matrigel revealed better RPE growth and notably less contamination with non-RPE cells than normal Matrigel or hESC-qualified Matrigel at similar dilutions.

Differentiated RPE cells can either be grown on normal cell culture surfaces or in 3D systems using filter membranes which allow polar growth of cells (Ukrohne et al. 2012; Zhu et al. 2013). The latter mimics a more natural situation and stimulates growth of native RPE cells (Strauss 2005). For example, we show that polar growth on transwell filters results in BEST1 protein expression at the basolateral plasma membrane, whereas growth on equally coated glass cover slips results in diffuse BEST1 protein staining with less efficient localisation to the plasma membrane. It should also be noted that in our experiments, culturing of hiPSC-RPE cells on transwell filter was possible for several months before cells started to die, whereas cells became apoptotic already after 6 weeks of cells cultured on equally coated cell culture plates. Cells could also successfully be detached enzymatically from filter membranes and further subcultured. Therefore, for hiPSC-RPE cells, we strongly prefer polarised growth on filter membranes.

Highly differentiated hiPSC-RPE monolayers can undergo serial expansion while retaining key cytological and functional properties for a number of passages before they reach replicative senescence (Singh et al. 2013a). Attempting to passage cultures beyond senescence has been demonstrated to greatly alter cellular characteristics. Thus, knowledge of this passage ceiling is important when preparing cultures for *in vitro* studies. It has also been emphasised that culture systems vary widely, and therefore, the optimal passage and expansion range must be determined for each culture method (Singh et al. 2013a). In our experiments, we also observed replicative senescence in hiPSC-RPE cell culture with alterations in cell morphology, BEST1 expression and TER anomalies, but generally not before passage 6 in very contrast to an earlier report where fundamental changes were already found at passage 3 (Singh et al. 2013a).

Typical hexagonal morphology of pigmented cells is unique for human RPE cells and on its own serves as an important indicator for cell identity (Buchholz et al. 2009; Singh et al. 2013a). Nevertheless, we set out to provide an in-depth characterisation of structural and functional aspects of hiPSC-RPE cells. Here, we also show by SEM that hiPSC-RPE cells develop the typical microvilli at their apical side. SEM analysis was performed with both low-vac and high-vac mode. While low-vac mode in moist conditions allows cells to better maintain their natural environment than critical point drying and high-vac mode, fluid on the cell surfaces, however, interfered with a clear presentation of microvilli. In contrast, high-vac mode and dry conditions fully revealed differentiation of the specialised apical microvilli essential for most functions of the native RPE, such as epithelial transport (Strauss 2005).

Our analysis also extended to immunostaining with BEST1 antibodies. This protein has been shown to be predominantly expressed in the human RPE, where it is specifically localised at the basolateral plasma membrane (Marmorstein et al. 2000; Stohr et al. 2002; Strauss 2005). Interestingly, this protein is susceptible to culturing conditions and usually stops expression within 5–6 days of primary cell culture growth. Our study now showed that high BEST1 expression was maintained for an extended period of time in cell culture and for many passages on transwell filters. In addition, both expression and localisation of BEST1 were comparable to native RPE cells when grown for at least 2 months in culture. We additionally confirmed BEST1 protein localisation by surface protein biotinylation which only labels plasma membrane proteins extending to the outside of the cell surface. Less than 2 months of cell culture revealed BEST1 immunostaining partly cytosolic with only some protein localised to the basolateral plasma membrane. This could indicate that hiPSC-RPE cells require several weeks of maturation for correct trafficking. When hiPSC-RPE cells finally reached replicative senescence at passage 6, not only their hexagonal cell shape was altered but also BEST1 protein was detected diffusely in the cytosol.

Formation of tight junctions is an essential feature of RPE cells and required to maintain the blood–retina barrier (Strauss 2005). Our results demonstrate presence of the tight junction marker ZO-1 (Stevenson et al. 1986) at the lateral apical side of hiPSC-RPE cells. This suggests formation of operating tight junctions necessary for correct RPE function and is also supported by a strong TER which was build up with time in cell culture and generally maintained at high levels in hiPSC-RPE cells. Replicative senescence at passage 6 still revealed hiPSC-RPE cells with regular ZO-1 labelling at the apical side, but TER measurement showed a slow decline. This is likely due to malfunctioning tight junctions and points to the validity of TER recordings as a key functional parameter.

Secretion properties are characteristic for native RPE (Strauss 2005) and have been previously described for hiPSC-RPE cells (Singh et al. 2013a), underlining the capacity of hiPSC-RPE to form functional, polarised monolayers. In accordance with these previous findings, we documented VEGF-A secretion in our hiPSC-RPE cell lines. VEGF-A quantity decreased with increasing passage, likely due to replicative senescence. As described previously, the majority of VEGF-A is secreted to the basal side where it acts on the choroidal endothelium (Strauss 2005; Singh et al. 2013a). Interestingly, we found higher apical than basal secretion. This is likely due to our coating method of filter inserts which might reduce basal VEGF-A amount and needs further investigation.

Phagocytosis of POS is another key function of RPE cells (Strauss 2005). In the present study, we verified phagocytotic properties of hiPSC-RPE cells in a timeline experiment and visualised the intracellular location and degradation of POS by immunofluorescence and confocal microscopy. Due to known autofluorescence of POS (Chang and Finnemann 2007; Lei et al. 2012), they were not only visible in the green channel, but also in the red channel. For statistical evaluation of POS incorporation, we only used images taken in the green channel. We observed a statistically significant increase in phagocytotic activity during the first 6 h. Between 6 and 24 h, there was a slight rise in the number of incorporated POS, although statistically not significant. This could be indicative of a saturation effect, beginning after 6 h of incubation with POS. Calculations indicate that after 24 h feeding with 20 POS per cell, each hiPSC-RPE cell should have incorporated two POS. In other words, 10 % of POS in the medium should be incorporated. As expected, we observed more partly digested and degraded POS which had lost their typical size and shape after 24 h than after 6 h of POS feeding.

Some of our experiments focused on long-term storage of hiPSC-RPE cells. For the purpose of establishing cell repositories of disease- or patient-specific cell lines, fibroblasts or hiPSC lines can be cryostored. Nevertheless, it would be more feasible to directly cryopreserve hiPSC-RPE as the target cells of interest. We therefore not only tested whether hiPSC-RPE cells were vital after cryopreservation, but also characterised their key structural and functional properties. Confocal microscopy verified BEST1 protein expression, but less efficiently localised to the basolateral plasma membrane compared to hiPSC-RPE cells without prior cryofreezing. Phagocytotic properties were not altered by cryopreservation. We directly compared continuously cultured cells to cryopreserved cells in the same experimental set-up and detected a generally lower phagocytotic activity after 24 h than in the timeline experiment. This underlines inter-experimental fluctuation and the importance of adequate controls. VEGF-A was secreted by cryostored hiPSC-RPE cells, but in lower amounts compared to continuous hiPSC-RPE cells. We moreover found a later but equally strong increase of TER in cryostored hiPSC-RPE cells, indicating a similar TER pattern as identified for continuous hiPSC-RPE cells. In conclusion, it seems best to use fresh, continuously cultured hiPSC-RPE cells, but cryopreserved hiPSC-RPE cells largely maintain key structural and functional aspects and can therefore serve as a reliable cellular resource.

In summary, our data demonstrate the successful reprogramming of human adult skin biopsy-derived fibroblasts to hiPSCs. We further established the generation of pure, expandable and cryostorable RPE cells from cultures of differentiating hiPSCs with high efficiency for further evaluation of structural and functional capabilities. In all aspects analysed, hiPSC-RPE cells were found to show structural and functional features characteristic for native RPE and thus hold tremendous potential as a tool for disease modelling in selected human ocular diseases with primary RPE pathology.

## Electronic supplementary material

Below is the link to the electronic supplementary material.

Supplementary material 1 (DOCX 128 kb)
