# Supplementary material for: In-Depth Characterisation of Retinal Pigment Epithelium (RPE) Cells Derived from Human Induced Pluripotent Stem Cells (hiPSC)
Source: Neuromolecular Med. 2014 May 7;16(3):551–64. doi: 10.1007/s12017-014-8308-8 (PMC4119585; doi:10.1007/s12017-014-8308-8)
Supplement: Supplementary file 1 — Supplementary material 1 (DOCX 128 kb) [file 12017_2014_8308_MOESM1_ESM.docx]

*
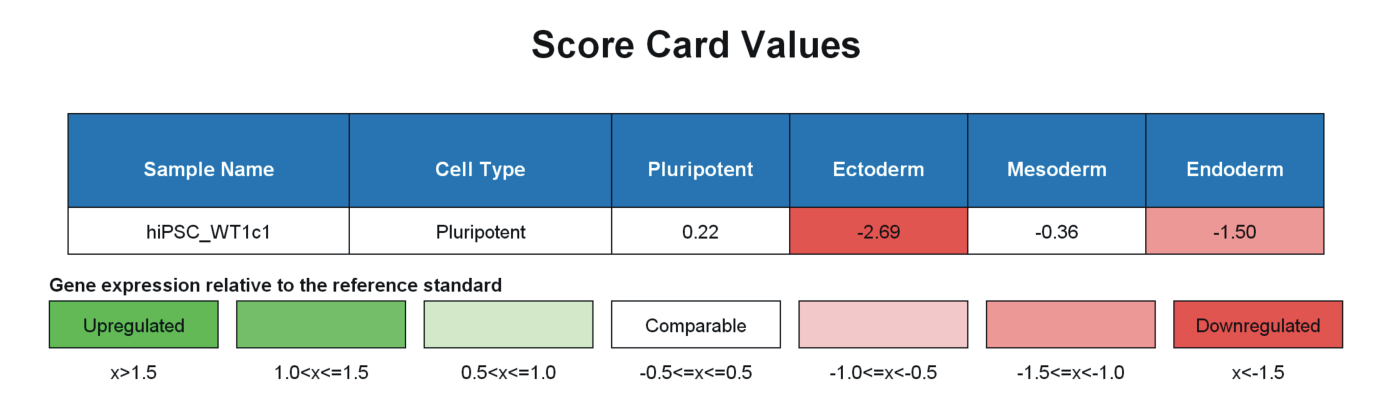
*

**Supplemental Figure S1. TaqMan hPSC Scorecard Panel evaluation of pluripotency**

Online data analysis software automatically assesses gene expression profiles relative to a reference standard and gives out automatically created value reports. Pluripotency marker expression of hiPSC_WT1c1 at passage 9 was comparable to the reference standard. With regard to germ layer markers, hiPSC_WT1c1 at passage 9 revealed downregulated expression values for Endoderm markers (-1.5 folds) and especially for Ectoderm markers (-2.69 folds) compared to the standard. Mesoderm markers were slightly downregulated but still comparable (-0.36 folds).

**Supplemental Table S1.** Gene IDs, oligonucleotide primer sets and sizes of amplification products for RT-PCR analysis

| **Gene** | **Primer Sequence (5` - 3`)** | **Product Size (BP)** | **GenBank Accession ID** |
| --- | --- | --- | --- |
| **OCT4** | Human ES/iPS Cell Pluripotency RT-PCR Kit (Applied Stem Cell, Menlo Park, USA) | 144 | NM_002701.4 |
| **SOX2** | Human ES/iPS Cell Pluripotency RT-PCR Kit (Applied Stem Cell, Menlo Park, USA) | 151 | NM_003106.2 |
| **NANOG** | Human ES/iPS Cell Pluripotency RT-PCR Kit (Applied Stem Cell, Menlo Park, USA) | 255 | NM_024865.2 |
| **TERT** | Human ES/iPS Cell Pluripotency RT-PCR Kit (Applied Stem Cell, Menlo Park, USA) | 114 | NM_198253.2 |
| **UTF1** | Human ES/iPS Cell Pluripotency RT-PCR Kit (Applied Stem Cell, Menlo Park, USA) | 408 | NM_003577.2 |
| **REX1** | Human ES/iPS Cell Pluripotency RT-PCR Kit (Applied Stem Cell, Menlo Park, USA) | 306 | NM_174900.3 |
| **DNMT3B** | Human ES/iPS Cell Pluripotency RT-PCR Kit (Applied Stem Cell, Menlo Park, USA) | 242 | NM_006892.3 |
| **COL1A1** | Forward: gggattccctggacctaaag | 319 | NM_000088.3 |
|  | Reverse: ggaacacctcgctctcca |  |  |
| **GAPDH** | Human ES/iPS Cell Pluripotency RT-PCR Kit (Applied Stem Cell, Menlo Park, USA) | 452 | AF261085.1 |
| **RPE65** | Forward: gcaaaattgtgcgcatctgc | 309 | NM_000329.2 |
|  | Reverse: cacccagatgccttggaaga |  |  |
| **BEST1** | Forward: cctgctgaacgagatgaaca | 328 | NM_004183.3 |
|  | Reverse: ggacacctgcaaattcctgt |  |  |
| **RLBP1** | Forward: acaagtatggccgagtggtc | 306 | NM_000326.4 |
|  | Reverse: ccacattgtaggtcgtggtg |  |  |
| **TYR** | Forward: ccatggataaagctgccaat | 347 | NM_000372.4 |
|  | Reverse: gcccagatctttggatgaaa |  |  |

*Primers were either acquired as a ready-to-use kit, or designed with Primer3.*

**Supplemental Table S2.** Antibodies and conditions used for immunofluorescence stainings

| **Primary Antibody** | **Manufacturer (Country)** | **Dilution** | **Secondary Antibody** | **Manufacturer (Country)** | **Dilution** |
| --- | --- | --- | --- | --- | --- |
| **Mouse anti human OCT4 monoclonal antibody (7E7)** | Pierce Antibodies (by Thermo Fisher Scientific, Schwerte, Germany) | 1:100 | **Alexa Fluor 488 goat anti-mouse IgG** | Invitrogen (by Life Technologies, Darmstadt, Germany) | 1:1000 |
| **Mouse anti human NANOG monoclonal antibody (1E6C4)** | Pierce Antibodies (by Thermo Fisher Scientific, Schwerte, Germany) | 1:250 | **Alexa Fluor 488 goat anti-mouse IgG** | Invitrogen (by Life Technologies, Darmstadt, Germany) | 1:1000 |
| **Rat anti human SSEA3 monoclonal antibody  (MC-631)** | Pierce Antibodies (by Thermo Fisher Scientific, Schwerte, Germany) | 1:500 | **Alexa Fluor 594 goat anti-rat IgG** | Invitrogen (by Life Technologies, Darmstadt, Germany) | 1:1000 |
| **Mouse anti human TRA-1-60 monoclonal antibody (ab129000)** | Abcam (Cambridge, United Kingdom) | 1:500 | **Alexa Fluor 488 goat anti-mouse IgG** | Invitrogen (by Life Technologies, Darmstadt, Germany) | 1:1000 |
| **Mouse anti human BESTROPHIN1 monoclonal antibody [E6-6] (ab2182)** | Abcam (Cambridge, United Kingdom) | 1:500 | **Alexa Fluor 488 goat anti-mouse IgG** | Invitrogen (by Life Technologies, Darmstadt, Germany) | 1:1000 |
| **Rabbit anti human ZO-1 polyclonal antibody (617300)** | Zymed Laboratories (by Life Technologies, Darmstadt, Germany) | 1:500 | **Alexa Fluor 594 goat anti-rabbit IgG** | Invitrogen (by Life Technologies, Darmstadt, Germany) | 1:1000 |
|  |  |  | **DAPI (4',6-Diamidino-2-Phenylindole, Dihydrochloride)** | Invitrogen (by Life Technologies, Darmstadt, Germany) | 1:1000 |
